# Supplementary material for: Motion model ultrasound localization microscopy for preclinical and clinical multiparametric tumor characterization
Source: Nat Commun. 2018 Apr 18;9:1527. doi: 10.1038/s41467-018-03973-8 (PMC5906644; doi:10.1038/s41467-018-03973-8)
Supplement: Supplementary file 1 — Supplementary Information [file 41467_2018_3973_MOESM1_ESM.pdf]

Supplementary Information for

"Motion Model Ultrasound Localization Microscopy for Preclinical and Clinical Multiparametric Tumor Characterization"

Opacic et al.

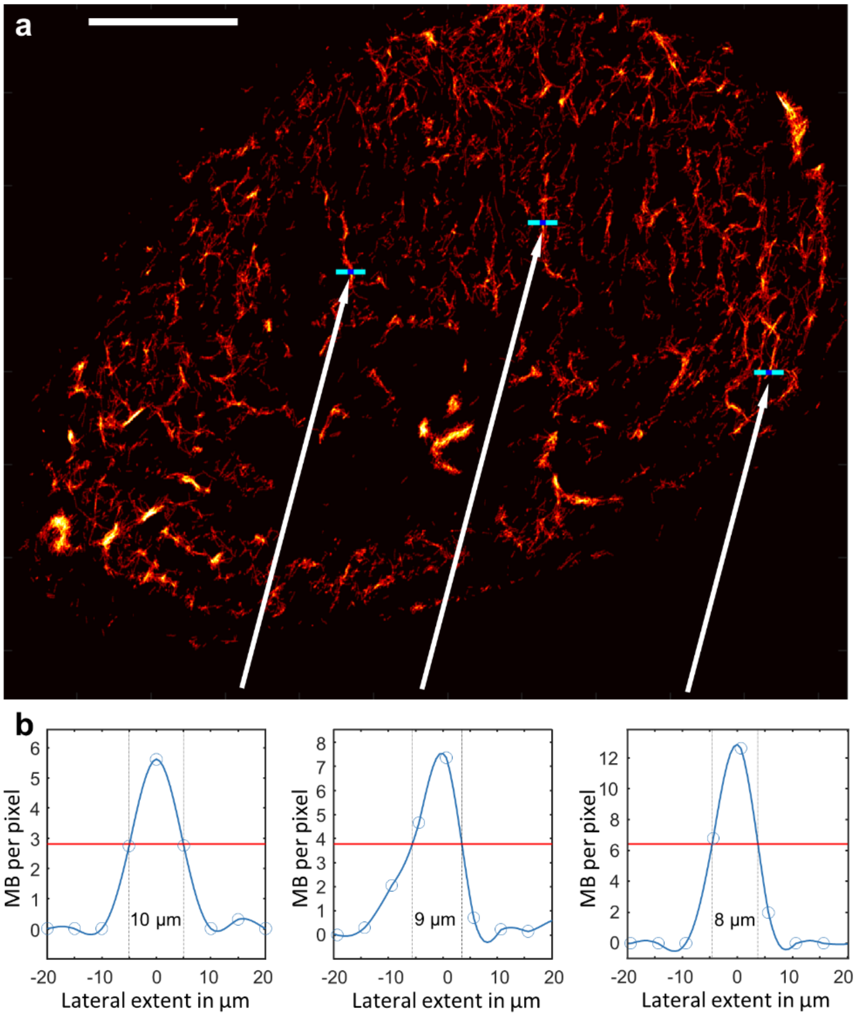

Supplementary Figure 1. Assessment of the spatial resolution obtained with mULM. The color-scaled image of microbubble counts per pixel of the A431 tumor shown in Fig. 2 was used. The image was rotated to align vessels predominantly in vertical direction. Three vessels with MB counts exceeding 5 events were horizontally cut as marked in the image (a). Scale bar, 1 mm. The dark blue center of the cyan bars cutting the vessels is the 40 µm cut that is displayed in the graphs below (b). The corresponding lines are interpolated by a factor of 8 to estimate the extent of the smallest vessels that were resolved. Vessel sizes measured as full-width-half-maximum were 8-10 µm exceeding the axial and lateral image resolution of the US system by a factor of 4 and 9, respectively. Small negative and non-integer values of MB counts in the cuts are caused by rotation and bandlimited interpolation. The original data resolution is indicated by the data-points (circles).

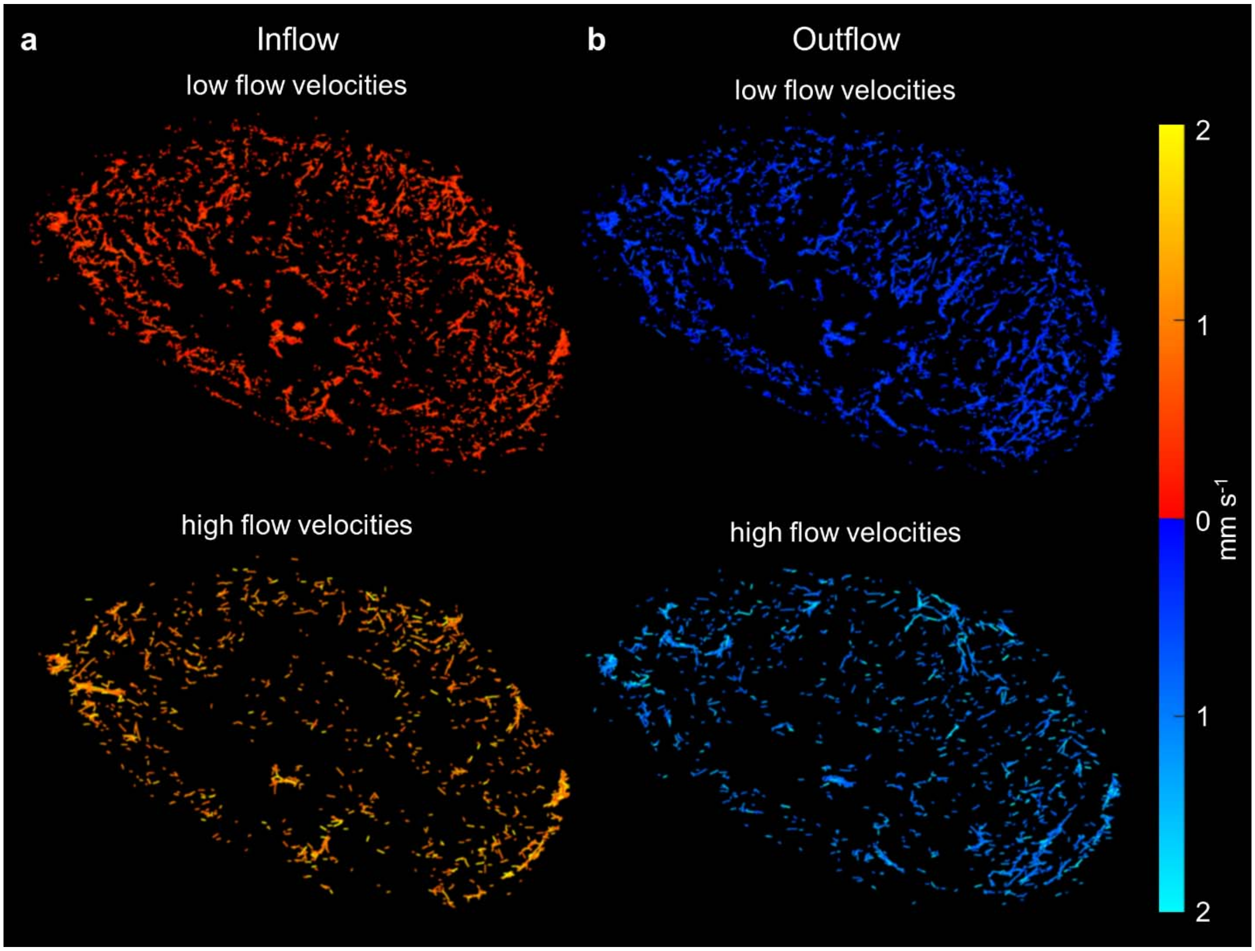

Supplementary Figure 2. mULM inflow and outflow direction maps in the A431 tumor. The maps illustrate the blood inflow depicted in red (a) and outflow displayed in blue (b). They show low (upper row) and high flow velocity vessels (bottom row) separately. For each track, the nearest point of the tumor border (applying the Euclidean distance transform: *bwdist* function, Matlab, MathWorks, Natick, MA, USA) was taken as the reference point. Then, the maps were computed by binarizing the flow directions into flow away from the reference point (inflow) and towards the reference point (outflow).

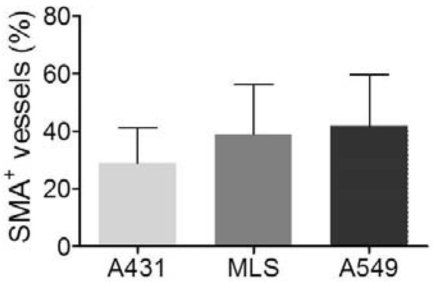

Supplementary Figure 3. Blood vessel maturity in tumors with different vascular phenotypes. The figure shows the differences in vessel maturity between the three tumor models as indicated by the percentage of smooth muscle actin (SMA) positive vessels. The overall degree of vessel maturity was highest in A549, followed by MLS and A431 tumors. Data are expressed as mean ± s.d. (*n*=4 per tumor model).

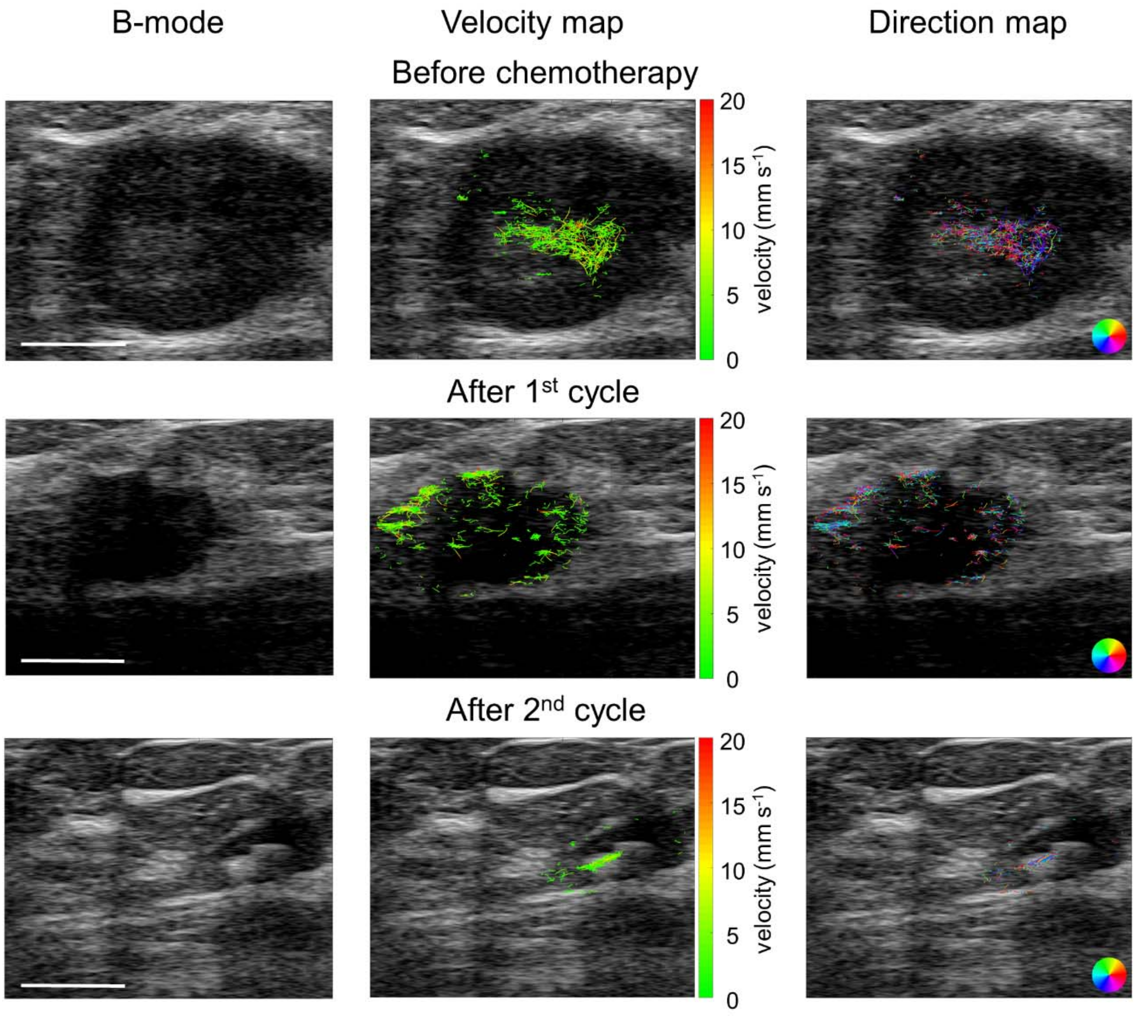

Supplementary Figure 4. B-mode and mULM images of a triple negative breast carcinoma in a patient treated with neoadjuvant chemotherapy. CEUS measurements were performed with a conventional US device and phospholipid MB before (first row), after the first (second row) and after the second cycle (third row) of chemotherapy. The first column shows B-mode images, the second column displays the mULM velocity maps and the third column indicates the mULM direction maps. Scale bars, 10 mm. At the baseline measurement the tumor displayed a low vascularity, and only in its center, the vascular networks were depicted without showing any dominant direction. After the first cycle of treatment, the tumor size had decreased, and vascularization appeared more homogeneous and more pronounced at the periphery. After the second cycle of treatment, the tumor had become very small with some functional vessels still being detectable.

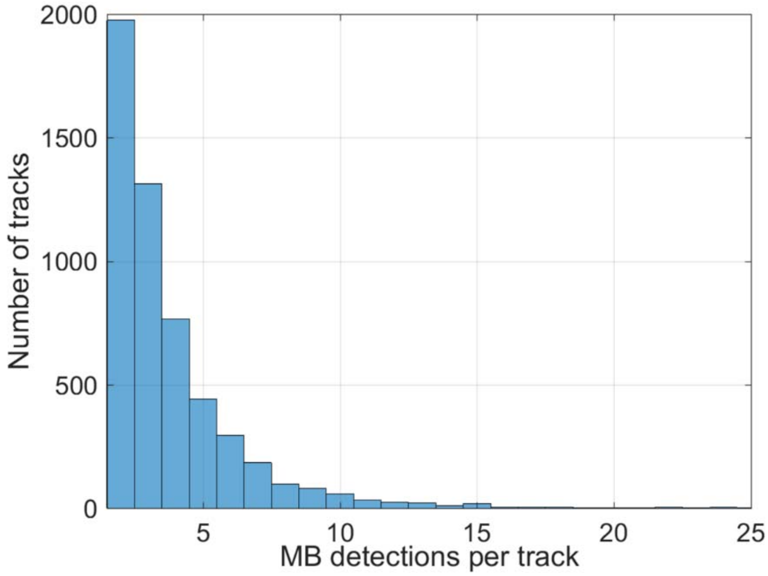

Supplementary Figure 5. Number of detections per track. The histogram shows the number of tracks over the number of detected MB assigned to these tracks for the mULM images of the patient with the HER2 positive breast carcinoma (see Fig. 6a). In the velocity and direction map of Fig. 6 only tracks of at least three associated detections were displayed.

	Ratios of areas of low / high flow velocitiy vessels	
	Inflow	Outflow
A431	1.81 ± 0.26	1.93 ± 0.24
MLS	1.66 ± 0.31	1.64 ± 0.19
A549	1.51 ± 0.18	1.57 ± 0.14

Supplementary Table 1. mULM inflow and outflow statistics. From the inflow and outflow maps of low and high flow velocity vessels (as shown in Supplementary Figure 2), the areas covered by the tracks were derived and the ratios of areas of low to high flow velocity vessels were calculated for the tumor types A431, MLS, and A549. The ratios for the inflow direction showed no distinct differences compared to the respective ratios for the outflow direction in tumor vasculature. This indicates that our threshold does not distinguish arteries and veins, which may be attributed to the fact that the majority of tumor vessels do not follow the typical vascular hierarchy and orientation of normal organs. Data are expressed as the mean ± s.d (*n*=4 per tumor model).

Categories of parameters		Parameter	CEUS postprocessing techniques		
			Destruction-Replenish. kinetics	Maximum Intensity Over Time	Motion Model ULM
Relative blood volume		Mean	magenta	yellow	turquoise
Local flow direction entropy		Mean	magenta	magenta	turquoise
Blood flow velocities		Mean	yellow	magenta	turquoise
		Variance	yellow	magenta	turquoise
		Maximum	yellow	magenta	turquoise
		Median	yellow	magenta	turquoise
Distances to the closest vessel		Mean	magenta	yellow	turquoise
		Variance	magenta	yellow	turquoise
		Maximum	magenta	yellow	turquoise
		Median	magenta	yellow	turquoise
Distances & velocities	Distances to vessel with low flow velocity	Mean	magenta	magenta	turquoise
		Variance	magenta	magenta	turquoise
		Maximum	magenta	magenta	turquoise
		Median	magenta	magenta	turquoise
	Distances to vessel with high flow velocity	Mean	magenta	magenta	turquoise
		Variance	magenta	magenta	turquoise
		Maximum	magenta	magenta	turquoise
		Median	magenta	magenta	turquoise

Supplementary Table 2. **Detailed overview of parameters obtainable with different CEUS postprocessing techniques**. Parameters are divided into five categories: 1) relative blood volume (rBV), 2) *local flow direction entropy*, 3) measurements of *blood flow velocities* (mean, variance, maximal and median) 4) measurements of the *distance to the closest vessel* (mean, variance, maximal and median) and 5) measurements of the *distances to vessels with low and high flow velocities*, respectively (mean, variance, maximal and median). The turquoise symbol indicates parameters that can be reliably obtained by the respective CEUS technique. The yellow symbol stands for the parameters which can only be obtained with low accuracy or not fully quantitative and the magenta symbol marks parameters, which cannot be calculated with the respective postprocessing CEUS technique.
